# Baseline Predictors of Sputum Culture Conversion in Pulmonary Tuberculosis: Importance of Cavities, Smoking, Time to Detection and W-Beijing Genotype

**DOI:** 10.1371/journal.pone.0029588

**Published:** 2012-01-04

**Authors:** Marianne E. Visser, Michael C. Stead, Gerhard Walzl, Rob Warren, Michael Schomaker, Harleen M. S. Grewal, Elizabeth C. Swart, Gary Maartens

**Affiliations:** 1 School of Public Health, University of the Western Cape, Cape Town, South Africa; 2 MRC Centre for Molecular and Cellular Biology, University of Stellenbosch, Cape Town, South Africa; 3 Division of Medical Microbiology, Institute of Infectious Diseases and Molecular Medicine, University of Cape Town, Cape Town, South Africa; 4 School of Public Health and Family Medicine, Centre for Infectious Disease Epidemiology and Research, University of Cape Town, Cape Town, South Africa; 5 Section of Microbiology and Immunology, The Gade Institute, University of Bergen, Haukeland University Hospital, Bergen, Norway; 6 Division of Dietetics, University of the Western Cape, Cape Town, South Africa; 7 Division of Clinical Pharmacology, Department of Medicine, University of Cape Town, Cape Town, South Africa; Fundació Institut d'Investigació en Ciències de la Salut Germans Trias i Pujol. Universitat Autònoma de Barcelona. CIBERES, Spain

## Abstract

**Background:**

Time to detection (TTD) on automated liquid mycobacterial cultures is an emerging biomarker of tuberculosis outcomes. The *M. tuberculosis* W-Beijing genotype is spreading globally, indicating a selective advantage. There is a paucity of data on the association between baseline TTD and W-Beijing genotype and tuberculosis outcomes.

**Aim:**

To assess baseline predictors of failure of sputum culture conversion, within the first 2 months of antitubercular therapy, in participants with pulmonary tuberculosis.

**Design:**

Between May 2005 and August 2008 we conducted a prospective cohort study of time to sputum culture conversion in ambulatory participants with first episodes of smear and culture positive pulmonary tuberculosis attending two primary care clinics in Cape Town, South Africa. Rifampicin resistance (diagnosed on phenotypic susceptibility testing) was an exclusion criterion. Sputum was collected weekly for 8 weeks for mycobacterial culture on liquid media (BACTEC MGIT 960). Due to missing data, multiple imputation was performed. Time to sputum culture conversion was analysed using a Cox-proportional hazards model. Bayesian model averaging determined the posterior effect probability for each variable.

**Results:**

113 participants were enrolled (30.1% female, 10.5% HIV-infected, 44.2% W-Beijing genotype, and 89% cavities). On Kaplan Meier analysis 50.4% of participants underwent sputum culture conversion by 8 weeks. The following baseline factors were associated with slower sputum culture conversion: TTD (adjusted hazard ratio (aHR) = 1.11, 95% CI 1.02; 1.2), lung cavities (aHR = 0.13, 95% CI 0.02; 0.95), ever smoking (aHR = 0.32, 95% CI 0.1; 1.02) and the W-Beijing genotype (aHR = 0.51, 95% CI 0.25; 1.07). On Bayesian model averaging, posterior probability effects were strong for TTD, lung cavitation and smoking and moderate for W-Beijing genotype.

**Conclusion:**

We found that baseline TTD, smoking, cavities and W-Beijing genotype were associated with delayed 2 month sputum culture. Larger studies are needed to confirm the relationship between the W-Beijing genotype and sputum culture conversion.

## Introduction

Tuberculosis cure after anti-tubercular therapy is best measured by bacteriological relapse within 2 years after completion of treatment. Because relapse is uncommon after short course therapy large sample sizes are required to detect differences in relapse rate, which, together with extended follow up periods, makes clinical trials using relapse as the primary endpoint very expensive. Sputum culture conversion after 2 months of treatment is a recognized surrogate biomarker of cure [1], [2]. Time to detection (TTD) on automated liquid mycobacterial cultures reflects mycobacterial load more accurately than sputum smear or culture grading, and has been shown to predict month 2 culture conversion, tuberculosis recurrence and relapse [3], [4].

Important factors associated with smear and culture non-conversion after 2 months include increasing age, chest radiographic features (cavitation and extent of disease), and higher sputum smear and culture grading at diagnosis [5]–[10].

There is limited evidence on the influence of *M. tuberculosis* genotype on the clinical outcome of tuberculosis. It has been suggested that the W-Beijing genotype has a selective advantage over other *M. tuberculosis* strains, since it is emerging worldwide and is linked to multidrug resistance in many areas [11], [12]. Data is conflicting regarding the role of the W-Beijing genotype on the risk of treatment failure or relapse [13]–[16].

We assessed baseline predictors, including W-Beijing genotype, of failure of sputum culture conversion within the first 2 months in participants with first episodes of smear positive pulmonary tuberculosis attending a primary care centre in Cape Town, South Africa.

## Methods

### Ethics statement

The trial protocol was approved by the University of Cape Town Ethics and Research Committee and written consent was obtained from all participants.

### Study population and setting

Participants with first episodes of sputum smear positive pulmonary tuberculosis were enrolled in a randomized controlled trial of a micronutrient intervention (vitamin A and zinc), which has been reported elsewhere [17]. In brief, adults who attended two primary care tuberculosis clinics in Delft, Cape Town, South Africa between May 2005 and August 2008 were recruited. Rates of poverty and unemployment are high in Delft and the tuberculosis incidence rate increased from 496 to 737 cases per 100 000 during the study period. Study participants received standard anti-tubercular therapy for 5 days a week consisting of combination tablets contributing 600 mg rifampicin, 300 mg isoniazid, 1.6 g pyrazinamide and 1.1 g ethambutol for participants weighing 38 to 55 kg (Rifafour, Aventis Pharma Pty Ltd, Johannesburg). Doses were adjusted for participants weighing less than 38 kg or more than 55 kg. All participants were on directly observed therapy at the clinic or in the community. Research staff collected early morning sputum specimens weekly from participants for 8 weeks. The micronutrient intervention had no effect on . time to sputum smear or culture conversion up to 8 weeks on Kaplan Meier analysis (*P* = 0.15 and *P* = 0.38 respectively; log rank test) The current analysis is restricted to trial participants with a positive baseline sputum culture and with data on susceptibility to rifampicin and isoniazid. Participants with rifampicin resistance during the 8 week follow-up period, were excluded.

### Study procedures and selection of isolates

In keeping with national guidelines at the time, none of our HIV-infected participants received anti-retroviral therapy, but all received co-trimoxazole prophylaxis. All participants were screened for misuse of alcohol with the Cut down, Annoyance, Guilt and Eye-opener (CAGE) questionnaire [18]. Smoking status was categorized as either never or ever smoked. Body weight and height of participants was determined. The extent and size of lung cavities at baseline was assessed independently by two pulmonologists experienced in the use of the Chest Radiograph Reading and Recording System (CRRS)- and disagreements on radiographic reading were resolved by consensus [19].

Prior to anti-tubercular therapy, trial participants supplied one spontaneous sputum specimen and thereafter one early morning unassisted sputum specimen every week, up to 8 weeks. Sputum specimens were examined by means of fluorescent microscopy (Auramine stain) and graded according to international standards [20]. All specimens were decontaminated and cultured on liquid media using the BACTEC MGIT 960 system (Becton Dickinson, Sparts, Maryland). Positive cultures were confirmed as *M. tuberculosis* complex using an in-house PCR assay [21]. The date of culture conversion was taken as the date of the first negative culture provided there were no subsequent positive cultures. Participants whose first negative culture occurred at week 8, were regarded as converters. Routine phenotypic drug susceptibility testing for isoniazid and rifampicin of isolates was carried out using the MGIT 960 system from January 2008; isolates cultured prior to that date were tested retrospectively, but some isolates had lost viability.

During 2009, spoligotyping was performed on the majority of serial isolates collected during the trial period [22]. Strain-specific PCR assays were performed to detect *M. tuberculosis* infections by W-Beijing and non-W-Beijing isolates [23]. In order to optimize the baseline data, we captured the strain type for all the available baseline isolates, as well as those within the first 2 weeks of treatment, in order to categorize participants as either W-Beijing or non-W-Beijing strain types. Ten participants who had two strain types, one of which was W-Beijing, were categorized as having the W-Beijing strain for the analysis.

### Statistical analysis

Data was captured in duplicate in Microsoft Office Excel 2003 and validated with SAS 9.2 software. To account for missing data, multiple imputation was conducted with the Amelia II software package [24],a reliable imputation procedure implemented in the statistical software package *R*,and five imputed data sets were generated. This number of data sets is generally considered adequate [25].

Cox proportional hazard regression modeling with the imputed datasets was used to estimate the hazard ratio of culture conversion for the following pre-determined baseline variables: age, gender, smoking status, alcohol abuse, sputum smear grading, sputum TTD, presence and extent of cavitation, *M. tuberculosis* genotype, HIV-infection, body mass index, haemoglobin, total lymphocyte count, serum C-Reactive Protein (CRP) (at baseline and change in concentrations within the first 2 weeks) and albumin. Hazard ratios are reported together with 95% confidence intervals based on Rubin's rules [25]. The proportional hazards assumption was verified by testing interaction effects of analysis time with baseline variables (α = 0.05) and graphically via log-log-plots [26].

To confirm the stability of our results we conducted a sensitivity analysis using Bayesian model averaging to each imputed data set (M = 20), and the results were combined by Rubins' rules [25], [27]. Model averaging is an alternative to model selection and combines estimates from different models, which contain different sets of variables, with the aim of achieving stable estimates that incorporate modelling uncertainty in addition to sampling uncertainty: Models that have a higher posterior probability to be correct receive a higher weight [26]. The combined weighted estimates can then be interpreted as a stabilized hazard ratio of culture conversion, which, together with adjusted standard errors, reflects the uncertainty in the modeling process. The posterior effect probabilities presented in our analysis state the probability that the hazard ratio in the Cox regression model for a variable is not one, thus providing a summary measure of how likely it is that a baseline variable has an effect and ranks the importance of the baseline variables.

## Results

### Baseline characteristics

**Figure 1:**
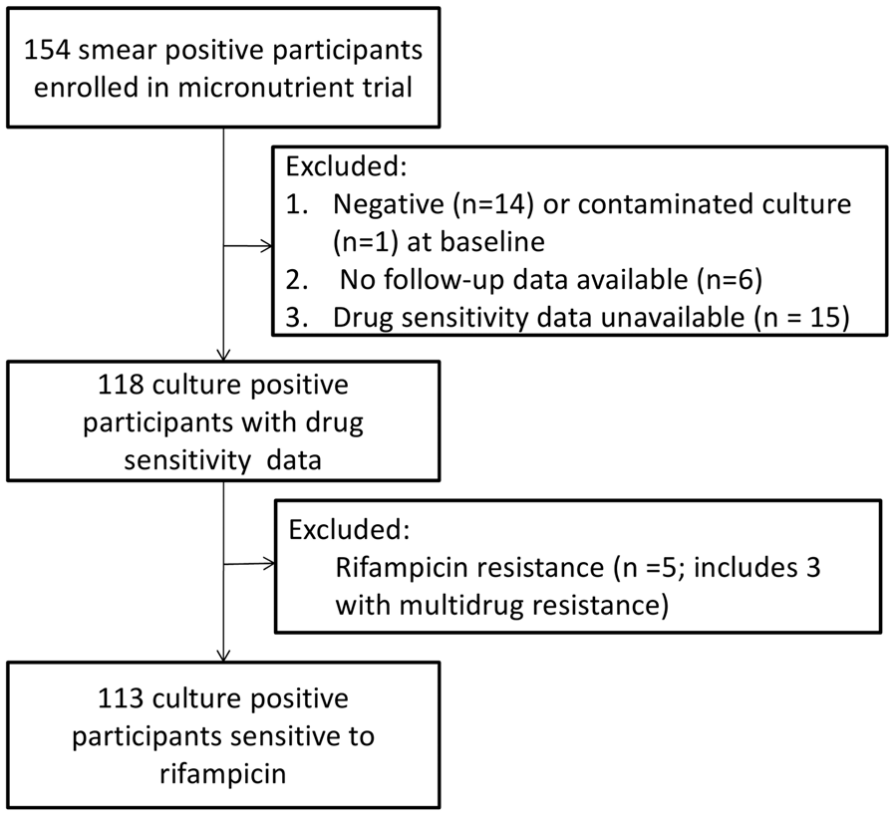
Participant selection from the parent micronutrient trial for the sputum culture conversion analysis.

A total of 154 participants with new smear-positive pulmonary tuberculosis were enrolled in the micronutrient trial, of whom 113 fulfilled the inclusion criteria for the current study (Fig. 1). Four of the 113 sputum isolates were mono-resistant to isoniazid. Complete data on all the variables investigated in the current analysis was available in 72 patients. The baseline characteristics of these participants before and after multiple imputation, is shown in Table 1. There were 11 HIV-infected participants (5 males, 6 females) and HIV-status was not determined in 9 who were lost to follow-up before the test could be done. All participants underwent chest radiograph examination at baseline, but 22 could not be located at the clinic site either due to mis-filing or loss (e.g. chest radiographs sent with participants who were referred to hospitals). Strain data was unavailable for seven participants whose sputum cultures were discarded before spoligotyping could be performed.

**Table 1:** Baseline characteristics of participants with drug sensitive smear-positive pulmonary tuberculosis.#

Characteristic	Before multiple imputation		After multiple imputation
	n		
Age, years	113	30.0 (22.0–43.0)	NA
Sex	113		
Female		34 (30.1%)	NA
Male		79 (69.9%)	
HIV-status	104		
Positive		11 (10.5%)	10.8% (95% CI:5.0; 16.6)
Negative		93 (89.4%)	89.2% (95% CI:83.4; 95.0)
Treatment group	113		
Micronutrient		56 (49.6%)	NA
Placebo		57 (50.4%)	
Sputum smear grade	113		
Scanty positive		1 (0.9%)	NA
1+		10 (8.9%)	
2+		13 (11.5%)	
3+		89 (78.8%)	
Time to culture detection, days	113	7.0 (6.0–11.0)	NA
*M. tuberculosis* genotype	106		
W-Beijing		50 (44.2%)	46.5% (95% CI:37.2; 55.9)
Other		56 (52.8%)	53.5% (95% CI:44.1; 62.3)
Chest radiograph	91		
Lung cavities		81 (89.0%)	88.1% (95% CI:82.1; 94.2)
No. of lung zones affected by cavities		2.0 (1.0–2.0)	1.8 (1.0–2.6)
Body Mass Index, (kg/m^2^)	112	18.9 (17.5–21.2)	18.9 (17.5–21.2)
Cigarette smoking	113		
Ever		85 (75.2%)	NA
Never		28 (24.8%)	
Alcohol misuse	113	43 (38.1%)	NA
Haemoglobin (g/dl)	111	12.1 (10.75–12.9)	12.03 (10.7–12.9)
Albumin (g/l)	111	36.9 (33.2–38.6)	36.86 (33.18–38.6)
C-Reactive protein (mg/l)	103	53.6 (36.4–75.8)	53.7 (36.6–75.8)
Total Lymphocyte count (×10^9^/l)	113	1.8 (1.35–2.24)	NA

# Continuous data expressed as median (IQR).

NA: Not applicable. Data was complete, therefore not imputed.

Participants had a high baseline bacillary burden, as reflected by the large proportion with a high sputum smear grading and a short median duration of TTD of 7 days (Table 1). Lung cavities were common and there was a high prevalence of smoking and alcohol abuse among participants. Spoligotyping showed that almost half of our participants had the W-Beijing strain type.

By week 6, nine percent of participants were lost-to follow-up and by week 8, this increased to 15%. Two cultures from participants who had not undergone culture conversion up to week 7, had contaminated cultures at week 8 and were categorized as not having converted. On Kaplan Meier analysis 50.4% of participants had undergone culture conversion by 8 weeks (data not shown).

### Predictors of delayed sputum culture conversion

Cox-proportional hazard regression analysis showed that TTD, the presence of lung cavities at diagnosis and ever smokers, were all associated with delayed culture conversion in univariate analysis. After adjustment for co-variates, TTD and lung cavities remained significant (Table 2). Although the W-Beijing genotype was not significant in univariate analysis, the confidence interval was suggestive of a delayed effect on conversion, even after adjustment for co-variates. INH mono-resistance was not associated with conversion in univariate analysis (HR = 0.59; 95%CI, 0.082; 4.28) and was not explored further due to the limited number of participants.

**Table 2:** Cox proportional hazards regression analysis of baseline variables associated with sputum culture conversion after multiple imputation*.

	Unadjusted Hazard ratio (95% CI)*	Adjusted Hazard ratio (95%CI)*	Posterior Effect Probability#
Age	0.99 (0.98–1.02)	0.98 (0.94–1.02)	1.1
Male sex	1.06 (0.57–1.97)	2.38 (0.88–6.25)	14.04
HIV-positive	1.62 (0.67–3.92)	0.65 (0.14–3.12)	0.43
Time to culture detection (days)	1.09 (1.03–1.16)	1.11 (1.02–1.2)	80.08
Sputum smear grading	0.79 (0.55–1.13)	0.75 (0.48–1.18)	10.08
Presence of lung cavities	0.32 (0.12–0.81)	0.13 (0.02–0.95)	87.61
No. of lung zones affected by cavities	0.92 (0.70–1.22)	0.99 (0.54–1.83)	9.52
W-Beijing genotype	0.62 (0.34–1.10)	0.51 (0.25–1.07)	41.24
Ever smoker	0.45 (0.25–0.82)	0.32 (0.1–1.02)	91.52
Alcohol misuse	1.13 (0.64–1.99)	1.67 (0.73–3.79)	10.97
Body Mass Index (kg/m^2^)	1.03 (0.94–1.13)	1.11 (0.96–1.31)	0.07
Haemoglobin (g/dl)	0.86 (0.71–1.03)	0.77 (0.58–1.03)	7.89
Albumin (g/l)	0.96 (0.90–1.03)	1.01 (0.89–1.15)	1.78
C Reactive Protein (CRP) (mg/l)	1.01 (0.99–1.01)	1.01 (0.99–1.02)	5.51
Change in CRP (baseline to week 2)	1.01(0.99–1.02)	0.99 (0.97–1.02)	0.98
Total Lymphocyte count (×10^9^/l)	0.86 (0.54–1.37)	0.81 (0.42–1.56)	5.42

*Likelihood of sputum clearance per unit change in predictor variable.

# Posterior effect probability after Bayesian Model averaging; this is the posterior probability that the Hazard in the Cox regression model for a variable is not one, taking model selection uncertainty into account.

The estimated hazard ratios of the variables remained similar after model averaging (estimates not shown) confirming the stability of our main results. After Bayesian model averaging ever smokers, the presence of lung cavities and TTD had a posterior effect probability of greater than 80%, thus having a rather strong effect on sputum conversion, even after taking model uncertainty into account. A moderate effect of W-Beijing genotype was also present (Table 2).

## Discussion

We found that a shorter baseline TTD, tobacco smoking, the presence of lung cavities and W-Beijing genotype were associated with delayed sputum culture conversion within the first 2 months in adults with smear-positive pulmonary tuberculosis. Bayesian model averaging showed that TTD, lung cavitation and smoking had strong effects and the W-Beijing genotype a moderate effect. The association we found between W-Beijing and sputum culture conversion has only been reported previously among a small number of participants, who participated in a multicentre trial [28]. Our finding that a shorter TTD is associated with delayed culture conversion adds to the very limited data on this potential surrogate marker [3], [4]. An increase of 3 days in TTD at baseline was associated with a 40% increase in the likelihood of conversion. Cavitation is a well- known factor associated with delayed sputum culture conversion [3], [5] and relapse [29].

Our data suggests that W-Beijing strains may be associated with delayed sputum culture conversion within the first 2 months, after adjusting for baseline bacillary load, the presence of lung cavities and the effect of smoking. Since the association between W-Beijing and culture conversion was largely unaffected by the adjustment for bacillary load at diagnosis and cavitatory disease, it suggests that other factors such as the host response to anti-tubercular treatment, may be important. Almost half of our participants had the W-Beijing genotype at baseline or within the first 2 weeks of treatment. The proportion of W-Beijing strains reported among adults and children with tuberculosis in Cape Town has ranged from 17 to 33% [30]–[32] and data from a paediatric referral hospital shows a marked increase in the prevalence of W-Beijing strains among children with tuberculosis between 2000 and 2003 [32]. One of the properties accounting for the rapid spread of the W-Beijing strains in our area may be operating by prolonging transmission in treated patients.

There is conflicting data on the role of smoking in sputum culture conversion [33]. Our unadjusted analyses showed that ever smokers had a significant longer time to culture conversion, compared with never-smokers during the first 2 months of treatment (HR = 0.45 (95% CI: 0.25–0.82)). This finding is similar to that of two other clinical trials [3], [34]. Retrospective data show that smokers are more likely to have clinical symptoms of tuberculosis such as cough and dyspnoea, as well as cavitary lesions, compared with non-smokers [35], [36]. When we adjusted for the presence of cavities and other confounders, the statistical significance of ever smoking was somewhat reduced, but smoking still had a strong effect in the final model, confirmed by model averaging estimates.

We were not able to demonstrate any significant effect of either baseline CRP concentrations, or change in concentrations after 2 weeks on treatment, on the risk of sputum culture conversion. CRP is a non-specific acute phase reactant and a marker of macrophage activation in active tuberculosis that is increased at diagnosis in proportion to the extent of disease and decreases during and after treatment [37]–[39]. Pre-treatment serum CRP concentrations have a high sensitivity for the diagnosis of active tuberculosis among smear-negative tuberculosis suspects [40] and was recently shown to be a risk factor for sputum smear conversion among participants in a micronutrient trial in Nigeria [9].

Our study has several limitations. First, there were missing observations in our data. We dealt with this by imputing missing data, which has been shown to be superior to complete case analysis (in which only subjects with no missing values are analyzed). If data is missing at random, it means that the missingness of data depends on observed co-variables or the outcome [41]. We find this assumption reasonable in our study, since the missing data related mainly to data capturing issues. Therefore, multiple imputation yields unbiased estimates that correctly reflect the uncertainty related to the missing data.

Second, a large proportion of our study participants did not convert during the 2 month period (49.6%), resulting in less statistical power. Similar high rates of culture positivity have been observed at 2 months at African sites using liquid culture media [4], [9], [42]. One of the strengths of our analysis is that we measured the time to sputum culture conversion, instead of a binary endpoint of culture conversion. Third, we did not assess the presence of a BCG scar among our participants. The absence of a BCG scar was recently shown to be an important factor in culture non-conversion among a Tanzanian cohort [8]. On the contrary, it has been suggested that the widespread use of the BCG vaccine may aid in the spread of W-Beijing strains, since it offers poor protection against W-Beijing strains in experimental models [11]. The BCG coverage among our local infants is high (99%) [43]. Fourth, rates of smoking and alcohol misuse were high in our study, but similar to those reported in another study from our region [4]. Variables associated with sputum conversion may be different in populations with lower rates of smoking and alcohol abuse.

Delayed sputum culture conversion increases the risk of relapse and prolongs the period of infectiousness. Therefore our findings about factors delaying culture conversion have several implications for public health. Smoking is associated with many health hazards, including increasing the risk of developing active tuberculosis [33]. Global tobacco use is increasing [44] and remains high in most countries with a high burden of tuberculosis [45], [46]. Effective tobacco control measures are needed, particularly in low-income countries, where smoking rates are highest among poorer and less educated communities [47]. Earlier detection of tuberculosis should reduce the number of patients presenting with cavitary disease and those with a higher sputum bacillary load and thus contribute to earlier sputum conversion. Finally, as mentioned above, BCG vaccination offers particularly poor coverage of W-Beijing strains. Newer tuberculosis vaccines will hopefully have greater activity against all strains, including W-Beijing.

In conclusion, a shorter baseline TTD, tobacco smoking, the presence of lung cavities, and W-Beijing genotype were associated with delayed sputum culture conversion within 2 months in adults with smear-positive pulmonary tuberculosis. Larger studies in high-burden settings are needed to confirm the relationship between the W-Beijing genotype and sputum culture conversion.
